# Bibliographical Notices

**Published:** 1856-09

**Authors:** 


### BIBLIOGRAPHICAL NOTICES.

*A Practical Treatise on the Diseases of the Testis and of the Spermatic Cord and Scrotum. With numerous wood engravings.* By T. B. Curling, F. R. S., Surgeon to the London Hospital, &c. *Second American from the second revised and enlarged English edition.* Philadelphia: Blanchard & Lea. 1856.

We are pleased to see another edition of the above excellent treatise. The author introduces it to the notice of the profession in the following terms: "More than twelve years," he says, "have elapsed since the publication of the first edition of this work. During that period, I have continued my enquiries into the morbid changes, which occur in the testicle, and have availed myself of increased opportunities of studying its diseases. In this edition some new chapters have been added; many have been re-written or altered; and, it is hoped, that nearly all of them contain additional facts of practical interest and importance.'

We have taken considerable pains to compare the present with the previous edition, and take pleasure in stating, as the result of our investigation, that it is in every way superior to it. In style and precision of language there is a marked improvement, a large amount of new and important matter has been introduced by the author, while his increased experience has enabled him to speak positively on many questions on which his opinions were indecisively or differently given in the first edition.

As it now stands, we can unhesitatingly speak of it as the first work upon the subjects it treats of in our language, one worthy in all respects of the confidence and approbation of the profession.

The first part of the American edition is taken up with the anatomical description of the scrotum and testis, to which is added a short account of the spermatic fluid. In the English edition, this part has been omitted for reasons stated by the author in his preface. "By a different typographical arrangement of the American edition," says the Publisher's advertisement, "space has been found for this valuable section without enlarging unduly the size of the work, and accordingly such portions of it have been retained, as had not been introduced by the author in various chapters throughout the volume."

Under the head of Diseases of the Testis, Chapter I. describes its various congenital imperfections and malformations under the sections of numerical excesses and defects, deficiencies and imperfection of the *vas deferens*, imperfect transition of the testicle and inversion of the testicle. In the section upon the imperfect transition of the testicle, will be found, a very clear and satisfactory description of the mode and agency by which the passage of the testicle into the scrotum is effected. "In some instances, the passage, though delayed, is completed at some period previous to puberty, and often a few weeks after birth." "My own observations," says Mr. Curling, "lead me to believe, that if the passage does not take place within a twelvemonth after birth, it is rarely fully and perfectly completed afterwards, without being accompanied with rupture. For the causes which operate at this late period, tend as much to promote the formation of hernia as the transition of the testicle. In cases where the testicle makes no appearance before puberty, uneasiness is often experienced at that period, owing to the enlargement of the gland being restrained by the rings and parts composing the inguinal canal. At the same time, also, it is often protruded outside the external ring by the movements of the abdomen in respiration." The causes of this failure of the testicle to pass into the scrotum, Mr. Curling attributes to deficient power in the *musculus testis*, to adhesions between the abdominal viscera, the result of peritonitis in the fœtus in utero, and to the smallness of the opening in the external abdominal ring, especially where the testicle is detained in the inguinal canal, in which situation, we are informed, it is more frequently found than in the cavity of the abdomen.

As practitioners are frequently liable to meet with these embarrassing cases, it becomes their duty to be acquainted with the proper methods of treating them. We feel, therefore, we cannot employ our space to better advantage than in copying Mr. Curling's excellent advice, the result of a large experience in such cases:—

"When a testicle is retained in the groin, there are various circumstances which tend to interfere with its evolution at puberty, to impede its nutrition and to excite inflammation and disease in it, and I have shown from dissections that such results are not unfrequent. A testicle, therefore, situated in the abdomen is in a more satisfactory position, and is much less exposed to injury and disease, than one which has been arrested in the groin. On this account, and as the passage is seldom perfectly accomplished when delayed beyond the age of one year, if the gland has not made its appearance at this period, the well being of the patient will be best consulted by the employment of some mechanical means to prevent the escape of the organ from the abdomen. A strong reason for adopting this practice is afforded by the great liability to rupture which exists in all cases of the tardy transition of the organ, owing to the persistence of a sac ready prepared for the reception of a protrusion, and in many instances to adhesions between the testicle and intestine or omentum. A hernia may occur whilst the testicle is still in the abdomen, or after it has passed the ring, and the viscera may descend into the scrotum, the gland being detained in the groin. Cases of this kind are embarrassing, as it is impossible to fulfil the two opposite indications of preventing the protrusion of the viscera, and encouraging the descent of the testicle. Many years ago I had under my care a fine child, neither of whose testicles had made their appearance out of the abdomen. When I first saw him, he was about a year old, and had an inguinal rupture on both sides, which descended whenever he cried or struggled. In accordance with the usual practice, I objected to the application of any truss. The parents became anxious and impatient at the annoyance arising from the hernia, and consulted a high authority, who gave similar advice to that received from me. The rupture was consequently left to itself, and the boy restrained from exercise. He was petted, became fretful, and proved a constant cause of uneasiness to the parents. When I last examined him he was eight years of age, and fortunately the rupture on the right side had disappeared spontaneously, and the one on the left protruded very slightly, but there was no appearance of the testicles. Now, if it be granted that a testicle situated in the abdomen is in a better position than one placed in the groin; that it is productive of less inconvenience, and exposed to fewer causes tending to impair its structure; that its subsequent passage, if it ever takes place, is frequently, if not commonly, attended with rupture, it must, I imagine, likewise be admitted, that the advice often given in these cases is unsound and injudicious. In recent years I have invariably advised the application of a truss so as to prevent the descent of the testicle as well as the escape of intestine, which I am sure has contributed much more to the health and comfort of the patient, than leaving him exposed to the inconveniences and dangers of an unrestrained rupture.

"In certain cases where the testicle has passed out of the external ring, but without descending fully into the scrotum, complicated with hernia, a truss with a small pad carefully applied may serve to keep up the rupture, and at the same time prevent the testicle from slipping back into the inguinal canal. When this can be done effectually without risk of the pad pressing on the testicle, it is the practice which should be adopted. But if the testicle is constantly gliding in the way of the pad so as to be exposed to pressure, or if adhesion exists between a portion of intestine and the gland, this treatment is inapplicable, and a truss should be applied to keep the parts if possible within the abdominal cavity.—A middle-aged gentleman consulted me on account of a large scrotal rupture on the right side. A great part, which consisted of bowel, could be returned without difficulty, but a mass remained irreducible unless in company with the testicle, and this was clearly made out to be a large portion of omentum adherent to the gland. On forcing up all the parts, I found it impossible to apply a truss without making pressure on the testicle, and more than ordinary pressure was needed to prevent the protrusion of so great a mass. So much inconvenience and risk attended leaving the rupture unrestrained, that I was compelled to apply a truss without returning the omentum, which was necessarily exposed to pretty strong compression from the truss-pad. The pressure led to his suffering occasionally from a dragging pain referred chiefly to the left side, particularly when he was affected with flatulency or distended bowels. The pain was relieved by easing the truss and rest in the recumbent posture. This gentleman had a varicocele on the left side, and wore a double moc-main lever truss, by which he was enabled readily to moderate the pressure."

Under the head of "Diagnosis in Cases of Imperfect Transition," besides mentioning the liability of the testis, when retained in the groin to be mistaken for a bubonocele, Mr. Curling adds, in the present edition, the following caution:—

"A testicle retained in the groin when inflamed, is liable to be mistaken for a bubo, the prominent oval swelling communicating a deceptive feeling of fluctuation, and being attended with pain; the skin over it occasionally exhibiting even a slight red blush, and the tumor being seated in a region where bubo constantly occurs and suppurates. It is related that Ricord, of Paris, was once very nearly deceived by a case of the kind, and even called for a knife to open the supposed abscess, but a re-examination of the tumor having led to the discovery of the absence of the testicle on that side of the scrotum, he made further investigation, and detected the true nature of the case."

The next two chapters treat of Atrophy of the Testicle and Injuries of the Testicle. Under the latter head, the author describes several curious cases of self-castration. These cases, it is stated, usually do well, the state of mind under which they were performed not generally operating prejudicially to the patient's recovery.

Hydrocele and its Palliative and Radical Cure, are fully discussed in Chapter IV. In Simple Vaginal Hydrocele, the superiority of injections over all other modes of treatment is strongly endorsed. Of the iodine injection the author speaks most favorably, and rarely resorts to any other :—

"The only apparatus required, in addition to a medium-sized trocar, is a half-ounce glass syringe with a metallic nozzle, which fits into a small stop-cock adapted to the canula. The metallic parts should be made of palladium, which is not acted on by iodine. I employed at first injections of the strength recommended by Mr. Martin (one drachm of the simple tincture of iodine to three of water), but I found this too weak, and I have used latterly a compound tincture of the following strength undiluted,—iodine ℈ij, iodide of potassium ʒss, spirits of wine ℥j,—injecting from two to three drachms, and allowing this to remain in the sac for five minutes. The greater part of the fluid is then withdrawn, about half a drachm only being left behind in the sac. Some surgeons are content to inject a drachm of the tincture, and to leave it in the sac, which answers quite as well. I have not found the tincture employed in this way in adults at all too stimulating. In operating, however, on persons under puberty, I dilute it one-half.

"I generally perform the operation on the patient standing, but it may be done equally well in the recumbent position. Directly the stimulating fluid becomes lodged in the vaginal sac, the patient generally feels sick and faint, and experiences pain in the part, and in the cord, with uneasiness in the loins. The pain is sometimes so severe that the removal of the injection becomes necessary before the expiration of the usual period. The amount of inflammation excited by the operation cannot, however, be estimated by the degree of pain suffered at the time. There is great difference in persons in their tolerance of stimuli, inflammation being more readily excited in some than in others, but its amount and intensity by no means depend on the susceptibility of individuals to pain."

It will be remembered that the previous edition advocated the use of lime water as the best fluid for injection.

Of the treatment by the seton, the author appears to entertain a more favorable opinion than he did at the date of the previous edition. In certain cases he even prefers it to the treatment by injection.

"In cases of simple hydrocele, after the failure of injections by others, I have also used the seton with success, and I have tried it, too, in cases where no other treatment has been adopted. The great objection to its use in simple hydrocele is the uncertainty of its operation. I have generally found it both a sure and gentle remedy, though occasionally I have been disappointed by its producing high inflammation, which it was impossible to control, and which speedily ran on to suppuration."

Hæmatocele, Orchitis, Tubercular disease, Carcenoma, Cystic-diseases, Fibrous and Cartilaginous Tumours of the Testis, Calcareous and other deposites, Entozoa in the Testicle, Sympathetic and Functional Disorder of the Testicle and Castration, are fully described in the succeeding chapters.

The second part of the work, comprising three chapters, is devoted to Diseases of the Scrotum. The various methods of treating varicocele, the most important of these affections, are fully described by Mr. Curling under the heads of palliative and radical treatment. Under the latter head, division of the spermatic vessels, the ligature, compression and excision, are severally discussed and generally disapproved of, as either being attended with risk of hæmorrhage or phlebitis, or liable to be followed by wasting of the testicle. The principal indication for the perfect cure of varicocele is to relieve the dilated and weakened veins of the weight of the blood lying in and above them, and "so enable them to return to their natural dimensions, and recover their tone so as duly to carry on the circulation." "The only plan," says Mr. Curling, in his first edition, "which appears to be fully adapted to effect this object, is firm, steady and continued pressure on the spermatic veins at the ring, by means of a well adjusted truss. At present, our experience of this mode of treatment is too limited to admit of any opinion of its efficacy being confidently expressed; but I look with no slight interest to the result of further trials of a remedy which seems to me to be based on sound views of the pathology of the disease."

The present work contains the author's further experience of the method, which, so far as we are informed, has proved in every instance in which it has been tried, remarkably successful, several cases being detailed by him in which the affection was entirely cured by pressure (by means of a moc-main lever truss), in from seven to nineteen months. "From these observations," Mr. Curling concludes, "it will appear, that I consider the treatment by pressure to be applicable either for the cure or relief of the majority of cases of varicocele occurring in practice. Certainly, in all those cases in which tolerably firm pressure with the fingers, at the abdomimal ring, removes the sense of weight and uneasiness along the cord, this plan may be resorted to with every prospect of a beneficial result; and its simplicity, freedom from all risk, and efficiency, render it preferable to all operative modes of treatment."

The remaining parts of the work are devoted to diseases of the Scrotum. We had marked several pages for extraction and comment in this portion of the work, but the space over which our article has already extended, obliges us to omit them.

The Publishers' advertisement states that the work has been passed through the press under the superintendence of Dr. Gobrecht, to whom we are also indebted for several additional engravings, and a case of interest. We are happy to say, that in reading the work pretty carefully, we have not detected a single typographical or other error in it. We observe, however, that the few notes introduced into the first edition by its American editor, although no notice whatever has been taken of them by the author in his second edition, are all of them again inserted in the present re-print. We mention the fact, not to disparage their value, but merely to state our opinion that it would have been better, under such circumstances, to have omitted them. The work, we should add, is handsomely printed and illustrated.

---

*Medical Jurisprudence.* By ALFRED S. TAYLOR, M. D., F. R. S., &c., &c. *Fourth American from the fifth and improved London edition. Edited with additions.* By EDWARD HARTSHORNE, M. D., &c. Philadelphia: Blanchard & Lea. 1856. 8vo. pp. 697.

We hazard little in affirming our belief that Taylor's Medical Jurisprudence is the best *manual* on its subject in any language. It has so long occupied the first rank among our most popular text books, and is so favorably known to readers of every kind, legal, medical and general, that the mere announcement of a new edition is an all-sufficient recommendation.

The previous efforts of its author and editor afford ample guaranty of continued improvements in each new issue of their work; and we need only to make a very cursory examination of the volume before us to be satisfied that the additions and alterations are both numerous and important, and such as fully to sustain the previous reputation of the manual, and that of its indefatigable author.

"Including the present edition," says Dr. Taylor in his preface to the fifth London edition, now some two years ago, "there have issued from the press, since the first publication of this work in November, 1843, ten thousand seven hundred and fifty copies. The encouragement that has thus been bestowed upon the author for his labors in a difficult branch of medical science, has induced him to revise with care the fifth edition now laid before the profession. The reader will find, on comparison, that considerable additions have been made, amounting in the whole to upwards of one hundred pages." It might be said, with equal truth, that similar encouragement in this country has stimulated editor and publishers to analogous efforts here, and the result is certainly a production which neither party need fear to compare with its predecessors from the same source.

The short interval which has elapsed since the publication of the English edition, and the evident desire of the American editor to confine his annotations altogether to that interval, have left the latter a very limited field of operation in the fulfilment of his duties. These accordingly have been, to use his own language, "principally confined to a careful revision of the text, the incorporation of the addenda, and the introduction of occasional brief notes of recent cases and decisions, and references to others as well as to some of the papers and works of interest which have been presented since the date of the author's preface." These notes and references are quite numerous, appear to be selected with care and discrimination, and cannot fail, we think, to add materially to the practical value of the book. They are concise, clear and to the purpose, and are so arranged as to add very slightly to the bulk of the volume, while they bring it very nearly up to the present date, and entirely up to that of its American preface.

We observe, in looking through the chapters, that several of the notes of the previous American editions have been incorporated and acknowledged by the author. This is a courtesy which, although natural enough, must be all the more gratifying and creditable to both parties, because it is not by any means the constant practice of some other equally prominent British authors, who at least appear to have accepted the hints of their American sponsors without seeming to be aware of any obligation. It is true that acknowledgements of this kind are more necessary and more likely to occur in a work that is so entirely made up of matters of fact gathered from every reliable source as a treatise on medical jurisprudence; still, we cannot help thinking our author's example in this instance worthy of note, as one which, in spite of the character of some of our volunteer editing, might, in all legitimate cases, be much more generally followed.

With regard to the improvements introduced by Dr. Taylor, we cannot serve our readers and the interests of the work better than by extracting from his preface, which he calls a "summary of the more important additions."

"Under Poisoning, numerous cases have been added, including new facts regarding the fatal doses of some of these agents, and the pathological changes which they produce. The subject of chronic poisoning has been more fully treated, and various improvements in the mode of applying tests for the detection of poisons have been introduced. In the chapters on Prussic Acid, Morphia, Strychnia, and Aconite, the reader will find some new cases which add to our knowledge of, and amend our experience on the operation of these poisons.

"Under Wounds, the following subjects have received additional illustration:—Ecchymosis,—the production of wounds by falls, and the method of distinguishing accident from homicide,—the influences of articles of clothing in modifying the appearance of personal injuries,—the direction of wounds as furnishing evidence of their origin,—the microscopical and chemical examination of clothes and weapons, especially in reference to the detection of blood, and the means which at present exist for distinguishing human from animal blood—the concealed causes of tetanus,—cicatrices from disease or wounds,—survivorship under severe wounds of the heart and injuries of the head,—ruptures of the liver, lungs, and bladder,—wounds from firearms as furnishing evidence of homicide,—the burning of the human body after death, and remarks on its alleged spontaneous combustion.

"Under Infanticide new cases will be found, which illustrate the causes of death in new-born children,—remarks on the medical evidence of the survivorship of the child, and the distinction between accidental and homicidal violence.

"The chapters on Pregnancy and Abortion have also received additions; and under Legitimacy, the medical evidence respecting gestation has undergone a full revision, by the aid of recently published facts and observations. It is unnecessary to specify the numerous additions which have been made to the remaining portions of the work. The chapters on Drowning, Hanging, Strangulation, and Suffocation, contain many additional facts, which may probably have an important bearing on medical evidence in future cases. Under Insanity, the provisions of the recent Acts of Parliament regarding medical certificates for the confinement of the insane, are fully described; and some further observations have been made in reference to the plea of insanity in criminal cases. Notices of many important trials (involving medico-legal questions) which have occurred in the United Kingdom from the date of the publication of the previous edition to the Lent Assizes of the present year, have been inserted in those parts of the volume to which the cases specially refer. In short, it has been the desire of the author throughout the whole of the work, to keep it up to the level of the day in regard to medico-legal information.

"In the preparation of this edition, the author has had the valuable assistance of the Right Honorable, the Lord Justice Clerk of Scotland. His Lordship has revised the whole of the sheets, and has not only furnished the author with many useful suggestions for the improvement of the work, but has enabled him to correct some errors which had crept into the reports of cases published in the earlier editions."

The student who is already familiar with the former edition, will be struck with many minor, but still useful changes, not enumerated in the author's preface. He will find also, that the index has been extended and rendered more complete, both as to topics and cases, and that the whole volume has been very carefully and neatly printed, and that, although presenting one hundred octavo pages of new matter, the work still retains its originally convenient form and size; that in short, it is more than ever an admirable specimen of a compact and comprehensive manual, which, although pretending only to the consideration of a hand-book and affording the facilities for one, yet possesses the weight and accuracy, if not all the fulness, of an authoritative treatise.

As such, we recommend it heartily to every one who may be interested,—and who, in these days of poisoning developements, is not?—in the fascinating details and discussions which belong to its prolific subject.

*Osteological Memoirs. No. 1. The Clavicle.* By JOHN STRUTHERS, M. D., Fellow of the Royal College of Surgeons of Edinburgh, Lecturer on Anatomy. Edinburgh: Sutherland & Knox. London: Simpkin, Marshall & Co. 1855.

Many of our readers will be surprised to learn that ninety pages have been devoted, by the author of the above memoir, to the description of a single bone, especially when we tell them that the consideration of the subject does not include its relations to pathology or comparative anatomy.

We are certain that most persons could exhaust their knowledge of the subject in a few paragraphs, and would hardly expect to find more than a few pages occupied with the peculiarity of one bone, even in a full text-book on Anatomy. How then, can there be in that single bone enough to interest even the author himself, is a question which might be readily asked.

An accomplished and thorough anatomist looks at a bone, not merely with reference to the general features by which it is recognised as a clavicle or sternum, but in it he sees peculiarities distinguishing it from other clavicles or other scapulæ. To him the ordinary description would be as barren as if you were to describe to a painter or ethnologist the figure of a man by merely indicating his general height, and the enumeration of his limbs, eyes, &c. To the anatomist every bone has its peculiarities. No two clavicles are precisely alike. As human countenances are varied to an unlimited extent, and yet possess the same features, so a bone to an anatomist presents, not merely a rough outline by its form, so that its position in the skeleton may be recognized, but to him it has also an expression peculiar to itself, if we may so speak, and which may even shadow forth the age, sex, strength and carriage of the living individual.

Mr. Struthers' descriptions are entirely from nature, and the minuteness of his detail, the carefulness of his measurements, and the fulness of his observation, show a laboriousness of research and patient investigation which must ever place him in an enviable position among his fellow anatomists.

He truly observes in the Preface:—

"Descriptive anatomy seems to be regarded by many as an exhausted science, but is far from being so. What science is, or can be so, when nature is the study and man the student? As Bacon has well said, 'They who confidently or magisterially pronounce of nature, as of a thing already discovered, have highly injured philosophy and the sciences.' Each new discovery, or method, or addition, opens the way to farther research and thought, and each new and greater application brings out new facts and principles, which gradually unfold themselves under the patient exercise of observation and thought, the combined use of the bodily and mental eye. I do not write thus of anatomy from enthusiasm; ten years in a dissecting room and at the lecture table afford ample time and occasion for mere enthusiasm to cool; but the longer I teach it, and the more I look into it, the more do I find it a rich field inviting new and farther investigation."

We can hardly expect the general reader, or a practitioner, to take much interest in the above memoir; but to those who are engaged in teaching anatomy or studying it as a science, we can recommend it as a valuable addition to their libraries.

---

*Physician's Tabulated Diary; designed to Facilitate the Study of Disease at the Bedside.* By A PHYSICIAN OF VIRGINIA. Richmond: J. W. Randolph. 1856.

The few efforts made as yet, to supply the want indicated by the above title-page, have hardly overcome all the difficulties it presents. This one, perhaps, succeeds as well as any other. It has the advantage, so far as real availability is concerned, in the brevity of its list of items to be recorded, and the consequent convenience of its bulk. There is, nevertheless, a corresponding disadvantage, greater, we think, than it need have been in this case, in the contracted space left for the record of most important particulars, *e. g.*, the symptoms and signs connected with the respiratory and circulatory apparatus, &c. We cannot but commend, however, the highly important purpose of its preparation, as stated to be, "the accumulation, at the bedside, of materials for analysis and generalization, which may, in time, elucidate questions of Medical Topography, Etiology, Therapeutics and Pathology."

